# Predicting Depression Risk in Physically Inactive Older Adults Using Dietary Antioxidants and Machine Learning: A SHAP‐Interpretable Analysis of NHANES

**DOI:** 10.1002/cns.70961

**Published:** 2026-05-30

**Authors:** Yuwen ShangGuan, Kunpeng Wu, Dong Li, Young‐Je Sim, Chuang Zhang, Zhenhao Lin, Litao Yan

**Affiliations:** ^1^ Changzhou Maternal and Child Health Care Hospital, Changzhou Medical Center Nanjing Medical University Changzhou China; ^2^ Department of Exercise Physiology Kunsan National University Gunsan Republic of Korea; ^3^ School of Physical Education and Health Zhaoqing University Zhaoqing China; ^4^ Department of Global Sports Industry Hanyang University Seoul Republic of Korea; ^5^ Department of Articular Orthopedics, The First People's Hospital of Changzhou The Third Affiliated Hospital of Soochow University Changzhou China

**Keywords:** depression, dietary antioxidants, elderly, machine learning, physical inactivity, SHAP

## Abstract

**Background:**

Physically inactive older adults represent a high‐risk group for depression. However, whether dietary antioxidant intake profiles can help stratify depression risk within this population has not been well established. This study aims to evaluate the predictive ability of dietary antioxidant intake for depression risk in physically inactive adults aged 60 and older using machine learning methods.

**Methods:**

This study utilized data from the 2007–2010 and 2017–2018 cycles of the National Health and Nutrition Examination Survey (NHANES), including 2,496 physically inactive adults aged 60 years and older. A total of 44 dietary antioxidants and two composite indices: The Composite Dietary Antioxidant Index (CDAI) and the Oxidative Balance Score (OBS) were assessed. Feature selection was performed using the random forest algorithm, followed by the development of six machine learning models: Random forest, XGBoost, k‐nearest neighbors, support vector machine, decision tree, and naïve Bayes. Model performance was evaluated using multiple metrics, including area under the receiver operating characteristic curve (AUC), accuracy, sensitivity, specificity, F‐beta score, and area under the precision‐recall curve (PR AUC). Ten‐fold cross‐validation and bootstrap resampling were employed to validate model robustness. Additionally, Shapley Additive Explanations (SHAP) analysis was conducted to facilitate individualized risk interpretation.

**Results:**

The random forest model demonstrated the best performance, with an accuracy of 94.9%, an ROC AUC of 0.943, and a sensitivity of 99.96%. SHAP analysis identified vitamin E, luteolin, total flavonoids, copper, magnesium, and iron as the most influential predictors, all of which showed a nonlinear inverse association with depression risk. Multivariable interaction analysis revealed synergistic protective effects between vitamin E and copper, as well as between luteolin and total flavonoids. In addition, an online risk prediction tool was developed based on the model, allowing for real‐time, personalized depression risk assessment upon input of key dietary antioxidant intake data.

**Conclusions:**

Dietary antioxidant intake demonstrated significant value in predicting depression risk among physically inactive older adults. Key nutrients and their interactions identified through machine learning and SHAP analysis provide new evidence and practical tools for targeted nutritional interventions and early screening of depression.

## Graphical abstract

This study applied machine learning and SHAP analysis to identify key dietary antioxidants that predict depression risk in physically inactive older adults, highlighting the roles of vitamin E, luteolin, flavonoids, copper, magnesium, and iron.

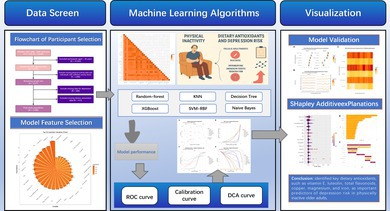

## 1 Introduction

With the accelerating pace of global population aging, mental health issues among older adults have become increasingly prominent [1]. According to the World Health Organization, approximately 280 million people worldwide suffer from depression. The prevalence among older adults is significantly higher than in other age groups, reaching 10%–20% in individuals aged 60 and above [2]. Late‐life depression severely impairs quality of life and social functioning and is closely associated with cognitive decline, worsening of chronic diseases, and increased mortality risk. Studies have shown that depression is a major risk factor for suicide among older adults, imposing a substantial burden on families and healthcare systems [3]. As population aging continues, the prevention and early identification of depression in older adults has emerged as a critical public health challenge worldwide.

Physical inactivity is highly prevalent among older adults, and more than 60% may fail to meet the World Health Organization's physical activity guideline [4]. A large body of epidemiological evidence indicates that lower physical activity is associated with a higher risk of depressive symptoms [5], whereas regular physical activity is linked to a ~20%–30% lower risk of depression in meta‐analytic evidence [6]. Given the high burden of inactivity in later life, identifying modifiable determinants and developing scalable, data‐driven approaches for depression risk stratification in this vulnerable subgroup is a public health priority.

A growing body of evidence suggests that oxidative stress plays a critical role in the pathophysiology of depression [7]. Due to age‐related physiological decline, older adults exhibit diminished endogenous antioxidant defenses, making them more susceptible to oxidative damage [8]. Oxidative stress can impair emotional regulation through multiple mechanisms, including disruption of neuronal membrane structure and function, interference with neurotransmitter synthesis and metabolism, activation of inflammatory signaling pathways, and damage to brain regions involved in mood regulation, such as the hippocampus and prefrontal cortex. Dietary antioxidants—such as vitamin E, vitamin C, flavonoids, luteolin, and anthocyanins—have the capacity to scavenge free radicals and alleviate oxidative stress. As a fat‐soluble antioxidant, vitamin E mitigates oxidative stress by neutralizing free radicals produced during lipid peroxidation, playing a vital role in neuroprotection. Flavonoid compounds, such as luteolin and total flavonoids, possess neuroprotective properties by suppressing neuroinflammation and modulating neurotransmitter metabolism. Moreover, minerals such as copper, magnesium, and iron serve as essential cofactors for antioxidant enzymes and are involved in neurotransmitter synthesis and energy metabolism. Numerous studies have reported that antioxidant‐rich dietary patterns are associated with a lower risk of depression, potentially through mechanisms involving neuroprotection, anti‐inflammatory effects, and regulation of neurotransmission [9].

Emerging epidemiologic evidence supports an association between higher dietary antioxidant exposure and fewer depressive symptoms in later life. Population‐based studies have reported inverse associations between dietary total antioxidant capacity and depressive symptoms, and analyses using composite antioxidant indices (e.g., the Composite Dietary Antioxidant Index, CDAI) have similarly suggested a lower likelihood of depression among individuals with higher antioxidant index levels [10]. Notably, most prior work has treated physical activity as a covariate rather than evaluating physically inactive older adults as a distinct high‐risk subgroup. Given that lower physical activity levels are consistently associated with a higher burden of depressive symptoms in older adults and that oxidative stress pathways are implicated in depression pathophysiology [11], examining dietary antioxidant profiles may be particularly informative for risk stratification in physically inactive older adults [12]. Although research on dietary antioxidants and depression has grown, important gaps remain. First, much of the existing literature focuses on single nutrients rather than capturing the combined and potentially non‐linear contributions of multiple antioxidant components within real‐world diets. Second, findings may vary across age groups and health contexts, and evidence specifically targeting physically inactive older adults is still limited. Third, even when predictive models are developed, many studies provide limited interpretability regarding how individual antioxidant‐related features jointly contribute to depression risk, which constrains translation into actionable nutritional insights. These limitations highlight the need for an interpretable multivariable framework to quantify the contribution of dietary antioxidant profiles to depression risk stratification in physically inactive older adults.

The application of machine learning techniques in nutritional epidemiology offers novel solutions to capture complex, potentially non‐linear relationships among multiple dietary components while improving predictive performance [13]. Nevertheless, evidence remains limited on interpretable, multivariable machine‐learning frameworks that leverage dietary antioxidant profiles to stratify the likelihood of current depression status among physically inactive older adults in population‐based settings. Therefore, using NHANES data, we aimed to develop and internally validate machine‐learning models to classify PHQ‐9–defined depression among physically inactive older adults based on dietary antioxidant–related variables and to use SHAP to quantify feature contributions and improve interpretability.

## 2 Methods

### 2.1 Data Source and Study Population

The National Health and Nutrition Examination Survey (NHANES) is a stratified, multistage probability sampling survey conducted by the National Center for Health Statistics (NCHS), which systematically collects data on participants' health status, dietary intake, physical activity, biochemical indicators, and sociodemographic characteristics. The survey protocol was approved by the NCHS Ethics Review Board, and all participants provided written informed consent. Detailed information on the NHANES study design and publicly available datasets can be accessed online at www.cdc.gov/nchs/nhanes/.

This study was based on data from three NHANES cycles: 2007–2008, 2009–2010, and 2017–2018, with an initial sample of 29,940 participants. Participants were sequentially excluded if they were under 60 years of age, had adequate physical activity, or had missing data on depression status or dietary antioxidant intake. A total of 2496 older adults were included in the final analysis. The study selection flowchart is presented in Figure S1.

### 2.2 Assessment of Dietary Antioxidant Intake

Data on the intake of 44 dietary antioxidants—including vitamins, minerals, and polyphenols—were obtained from NHANES. Participants completed the first 24‐h dietary recall interview in person at the mobile examination center (MEC), and a second 24‐h recall was collected by telephone 3–10 days later. The average daily intake of each antioxidant was calculated based on these two recalls. To comprehensively assess the predictive potential of dietary antioxidant profiles for depression, two composite indices were calculated: The Composite Dietary Antioxidant Index (CDAI) and the Oxidative Balance Score (OBS). Detailed calculation methods are provided in Table S1.

### 2.3 Definition of Physical Inactivity

Physical activity (PA) indicators in this study were primarily derived from self‐reported questionnaire data in the NHANES survey. The questionnaire collected information on vigorous work activity, moderate work activity, transportation‐related physical activity (TPA; walking and cycling), vigorous recreational activity, and moderate recreational activity. It also recorded the frequency of PA sessions and their duration over the past week [14, 15]. Exercise intensity is typically expressed in metabolic equivalents (MET), which represent the ratio of energy expended during PA to the energy expended at rest [16]. MET values vary by type of activity, and NHANES provides recommended MET values for each PA type. The calculation formula is as follows: PA (MET‐minutes/week) = MET value × duration per session × frequency per week [17]. MET‐minutes/week was used as the quantitative measure of physical activity intensity. According to the World Health Organization (WHO) guidelines on physical activity, a total weekly PA volume of less than 600 MET‐minutes/week is classified as insufficient physical activity [14]. To ensure the representativeness and accuracy of the sample, individuals with missing PA data or with a total MET score exceeding 600 MET‐minutes/week were excluded. All participants included in the final analysis met the criteria for insufficient physical activity.

### 2.4 Depression Assessment

Depressive symptoms were assessed using the 9‐item Patient Health Questionnaire (PHQ‐9), a widely used international screening tool for depression that consists of nine items, with a total score ranging from 0 to 27 [18]. The PHQ‐9 was administered during the MEC private interview in the same NHANES survey cycle as the dietary assessment. According to the PHQ‐9 scoring criteria [19], a total score greater than 9 was used as the threshold for identifying the presence of depressive symptoms, corresponding to the clinical standard for moderate or more severe depression [20]. In this study, a PHQ‐9 score > 9 was used to define depression status as a binary variable, and all included participants completed the PHQ‐9 assessment. Depression status served as the target variable in this study and was used for model development and performance evaluation. For specific scoring criteria, see Table S2.

### 2.5 Candidate Predictor Variables

Candidate predictors comprised dietary antioxidant–related variables (including individual antioxidant intakes and the two composite indices, CDAI and OBS), as well as baseline covariates available in NHANES, including age, sex, race/ethnicity, poverty–income ratio (PIR), marital status, education level, smoking status, alcohol use, body mass index (BMI), and medical history variables (hypertension, diabetes, hyperlipidemia, cardiovascular disease, stroke, cancer, and chronic kidney disease).

### 2.6 Preprocessing of Machine Learning Features

Prior to model development, comprehensive feature preprocessing was conducted to enhance model performance and address potential data‐related issues. First, out of 66 initial features, the random forest algorithm was employed to identify the top 30 most predictive variables. To reduce feature redundancy, Pearson correlation coefficients were calculated among the selected variables, and highly correlated features (*r* > 0.9) were removed to minimize collinearity and ensure independent contributions. To address class imbalance, the Synthetic Minority Oversampling Technique (SMOTE) was applied to generate synthetic minority samples and balance the class distribution, thereby reducing model bias toward the majority class. Additionally, normalization and standardization were applied to ensure all features were on a comparable scale, preventing features with larger ranges from dominating the model. These preprocessing steps resulted in a high‐quality, balanced, and non‐redundant feature set, laying a solid foundation for subsequent model training and significantly improving model stability and predictive performance.

### 2.7 Statistical Methods

Within the mlr3 framework, six machine learning models were developed, including XGBoost, decision tree, naive Bayes, k‐nearest neighbor (KNN), random forest, and support vector machine with a radial basis function kernel (SVM‐RBF). To comprehensively evaluate model performance, multiple metrics were used, including classification error rate, accuracy, F‐beta score, area under the receiver operating characteristic curve (ROC AUC), sensitivity, specificity, and area under the precision‐recall curve (PR AUC). Ten‐fold cross‐validation was applied for resampling to minimize evaluation bias. Differences in model performance were compared using analysis of variance (ANOVA) and the Kruskal–Wallis H test. To interpret feature importance in the best‐performing model, the SHAP method was employed. SHAP, grounded in cooperative game theory, quantifies the marginal contribution of each feature to individual predictions, providing insights into both global model behavior and personalized prediction explanations. All analyses were conducted using R software (version 4.4.1), with key packages including mlr3, mlr3learners, mlr3extralearners, mlr3benchmark, kernelshap, shapviz, DMwR, ggcor, ggplot2, and survey.

## 3 Results

### 3.1 Baseline Characteristics of the Population

A total of 2496 participants were included in this study, 221 of whom suffered from depression. Compared with non‐depressed participants, those with depression had significantly lower intakes of β‐carotene (1775.87 [2198.75] vs. 2216.83 [2908.82]), carotene retinol equivalent (161.45 [201.33] vs. 201.54 [265.13]), vitamin C (64.35 [59.41] vs. 77.29 [64.76]), vitamin E (6.06 [3.69] vs. 6.75 [4.09]), and iron (11.97 [5.34] vs. 13.07 [6.28]). For more information on dietary antioxidants, see Table S3. In addition, the depressed group had lower values in both the Composite Dietary Antioxidant Index (CDAI: −0.97 [3.00] vs. –0.29 [3.47]) and the Oxidative Balance Score (OBS: 16.33 [6.33] vs. 17.47 [6.76]). Significant differences were also observed between groups in body mass index, racial composition, marital status, educational attainment, smoking and alcohol use behaviors, as well as the prevalence of comorbid conditions such as cardiovascular disease and stroke. Detailed baseline characteristics are presented in Table 1.

**TABLE 1:** Baseline characteristics of the participants.

Variable	Non‐depression (*N* = 2275)	Depression (*N* = 221)	*p*
**Gender, *n* (%)**			0.027
Male	984 (43.3)	78 (35.3)	
Female	1291 (56.7)	143 (64.7)	
**Race, *n* (%)**			0.027
Non‐hispanic white	1175 (51.6)	99 (44.8)	
Non‐hispanic black	517 (22.7)	43 (19.5)	
Mexican american	276 (12.1)	40 (18.1)	
Other hispanic	204 (9.0)	26 (11.8)	
Other race	103 (4.5)	13 (5.9)	
**Marital, *n* (%)**			0.006
Married/living with partner	1291 (56.7)	101 (45.7)	
Widowed/divorced/separated	888 (39.0)	107 (48.4)	
Never married	96 (4.2)	13 (5.9)	
**Education, *n* (%)**			0.003
Below high school	371 (16.3)	53 (24.0)	
High school	943 (41.5)	96 (43.4)	
Above high school	961 (42.2)	72 (32.6)	
**Smoke, *n* (%)**			0.002
Never	1133 (49.8)	99 (44.8)	
Former	858 (37.7)	76 (34.4)	
Now	284 (12.5)	46 (20.8)	
**Alcohol, *n* (%)**			0.015
Never	425 (18.7)	47 (21.3)	
Former	575 (25.3)	72 (32.6)	
Now	1275 (56.0)	102 (46.2)	
**Hypertension, *n* (%)**			0.330
No	1708 (75.1)	173 (78.3)	
Yes	567 (24.9)	48 (21.7)	
**Diabetes, *n* (%)**			0.051
No	1183 (52.0)	102 (46.2)	
Yes	1065 (48.0)	119 (53.8)	
**Hyperlipidemia, *n* (%)**			0.490
No	1899 (83.5)	189 (85.5)	
Yes	376 (16.5)	32 (14.5)	
**CVD, *n* (%)**			< 0.001
Yes	590 (25.9)	87 (39.4)	
No	1685 (74.1)	134 (60.6)	
**Stroke, *n* (%)**			0.016
Yes	235 (10.3)	35 (15.8)	
No	2040 (89.7)	186 (84.2)	
**Cancer, *n* (%)**			0.383
Yes	474 (20.8)	40 (18.1)	
No	1801 (79.2)	181 (81.9)	
**CKD, *n* (%)**			0.188
Yes	985 (43.3)	85 (38.5)	
No	1290 (56.7)	136 (61.5)	
CDAI	−0.29 (3.47)	−0.97 (3.00)	0.005
OBS	17.47 (6.76)	16.33 (6.33)	0.016
PIR	2.46 (1.44)	1.91 (1.28)	< 0.001
BMI, kg/m^2^	29.98 (6.64)	30.97 (6.67)	0.034

*Note:* Continuous variables were presented as medians and standard deviations (SD), while categorical variables were described using frequencies and percentages.

Abbreviations: CDAI, Composite Dietary Antioxidant Index; CKD, Chronic Kidney Disease; CVD, Cardiovascular Disease; OBS, Oxidative Balance Score; PIR, Poverty Income Ratio.

### 3.2 Model Feature Selection

**FIGURE 1:**
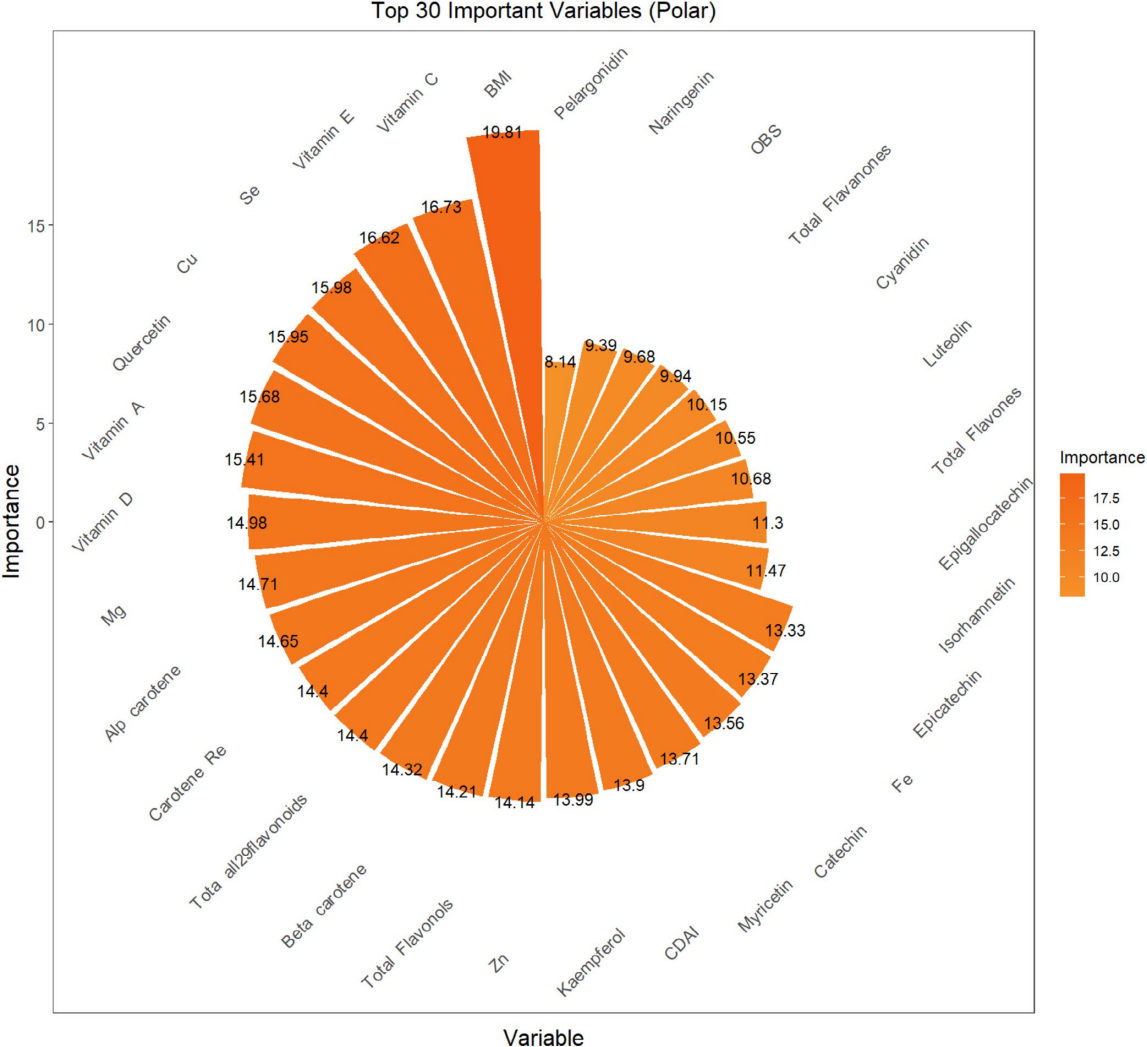
Top 30 features based on importance for PHQ‐9 prediction.

**FIGURE 2:**
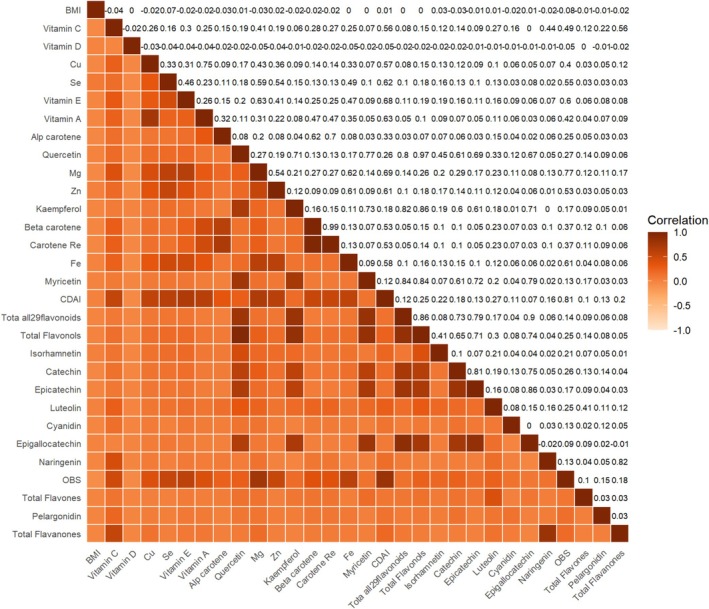
Heat map of correlations between random forest screening features.

A total of 66 variables were initially included at the model construction stage. Feature importance was evaluated using the random forest algorithm, and the top 30 most predictive variables were selected for model development (Figure 1). To reduce the potential impact of multicollinearity on model performance, Pearson correlation coefficients were calculated between selected variables. As shown in Figure 2, several dietary antioxidant features exhibited high intercorrelation, such as Carotene_Retinol_Equivalent, Beta_carotene, Quercetin, and Total_Flavonols. The final machine learning model included 25 dietary antioxidant features and one baseline characteristic.

### 3.3 Model Development and Performance Comparison

Six machine learning models were developed to predict depression risk among physically inactive older adults, including random forest, XGBoost, k‐nearest neighbors (KNN), support vector machine with radial basis function kernel (SVM‐RBF), decision tree, and naive Bayes. Model performance was evaluated using multiple metrics, including classification error rate, accuracy, F‐beta score, area under the ROC curve (AUC), sensitivity, specificity, and area under the precision‐recall curve (PR AUC). As shown in Table 2, performance metrics differed significantly among the models (all *p* < 0.001). The random forest model outperformed others, achieving the lowest classification error rate (0.0510), highest accuracy (0.9490), F‐beta score (0.9698), and AUC (0.9425). It also attained the highest sensitivity (0.9996) and a relatively high specificity (0.7181). Importantly, it achieved the highest PR AUC (0.9180), indicating a strong ability to detect positive cases in imbalanced data. The XGBoost model ranked second, with an AUC of 0.9230 and a PR AUC of 0.8791, though its specificity was relatively low (0.4726). The KNN model demonstrated a moderate and balanced performance, with a sensitivity of 0.8996, specificity of 0.7343, and PR AUC of 0.6283. In contrast, SVM‐RBF (PR AUC = 0.4996), decision tree (0.4820), and naive Bayes (0.3139) showed poor performance in PR AUC, indicating limited ability to identify individuals with depression. Although some models (e.g., SVM‐RBF with a sensitivity of 0.9667) demonstrated acceptable sensitivity, their extremely low specificity may lead to a high false positive rate, thereby limiting practical application.

**TABLE 2:** Comparison of the performance of six machine learning models.

Model	Classification error	Accuracy	F‐beta	AUC (ROC)	Sensitivity	Specificity	Area under the PR curve
Random forest	0.0510	0.9490	0.9698	0.9425	0.9996	0.7181	0.9180
XGBoost	0.0966	0.9034	0.9443	0.9230	0.9978	0.4726	0.8791
K‐nearest neighbors	0.1301	0.8699	0.9190	0.8703	0.8996	0.7343	0.6283
SVM (RBF)	0.1549	0.8451	0.9110	0.7577	0.9667	0.2901	0.4996
Decision tree	0.1597	0.8403	0.9100	0.7377	0.9840	0.1846	0.4820
Naive bayes	0.1874	0.8126	0.8940	0.6997	0.9636	0.1237	0.3139
*p*‐value	< 0.001^a^	< 0.001^a^	< 0.001^a^	< 0.001^b^	< 0.001^a^	< 0.001^a^	< 0.001^a^

Abbreviation: SVM, Support Vector Machine.

^a^ ANOVA test.

^b^ Kruskal‐Wallis.

**FIGURE 3:**
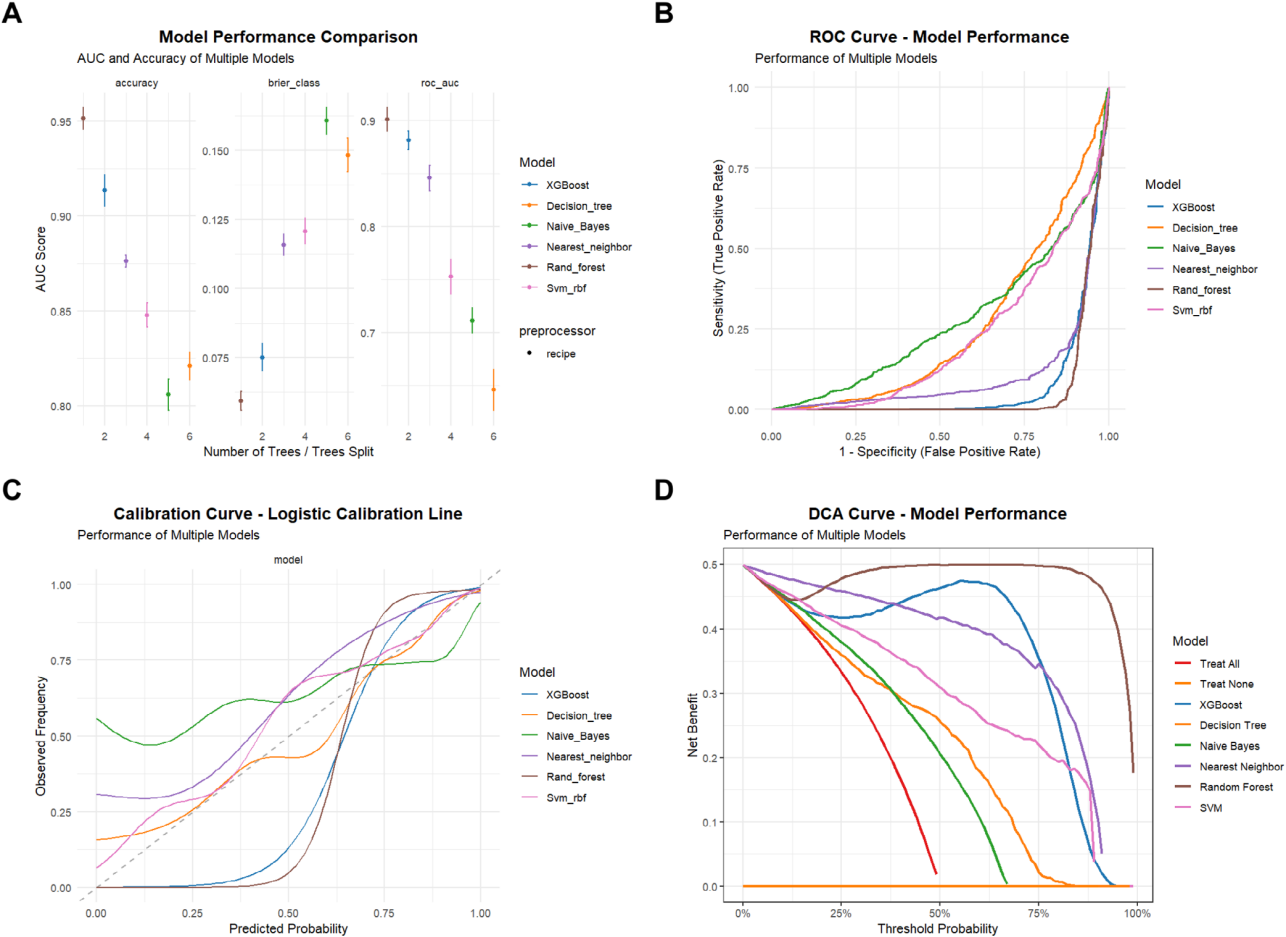
Performance assessment of six machine learning models in predicting depression risk in physically inactive older adults. (A) Comparison of the performance of different models in terms of AUC, accuracy, and Brier scores; (B) ROC curves of each model; (C) calibration curves of each model, assessing the agreement between predicted probabilities and actual observations; and (D) Decision Curve Analysis (DCA), reflecting the net clinical benefit at different probability thresholds.

Figure 3 provides a detailed visualization of model performance. The random forest model demonstrated the best performance on the ROC curve (Figure 3B) and showed good agreement between predicted and observed outcomes in the calibration curve (Figure 3C). In the decision curve analysis (Figure 3D), this model consistently yielded the highest net benefit across a wide range of probability thresholds, supporting its potential utility in clinical decision support for depression screening.

### 3.4 Model Validation

**FIGURE 4:**
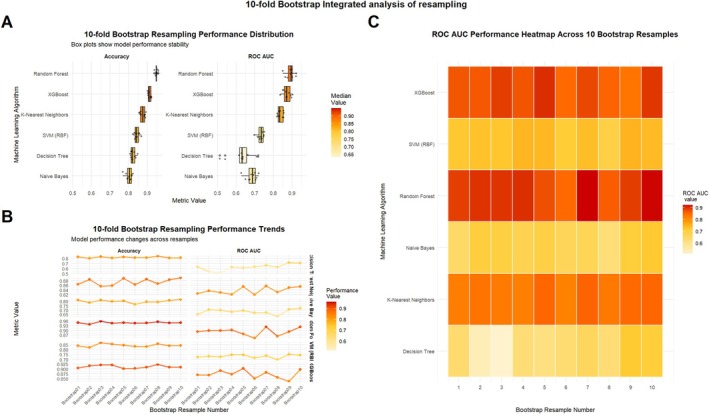
Validation performance of six machine learning models in 10 Bootstrap resamplings. (A) Distribution of accuracy (Accuracy) and area under the ROC curve (AUC) in 10 Bootstrap resamplings. (B) Trend plot of performance in resampling. (C) Heatmap of ROC AUC in 10 Bootstrap samples.

To assess the stability and generalizability of the models, we performed evaluation using 10‐fold cross‐validation combined with 10 bootstrap resampling iterations for each of the six machine learning models. As shown in Figure 4A, the random forest model demonstrated the highest median values and lowest variability in both accuracy and AUC, indicating superior stability. XGBoost followed closely, while KNN exhibited moderate performance with acceptable variability. Figure 4B further illustrates the performance trends across different resampling iterations. The performance of the random forest and XGBoost models remained consistent, whereas SVM, decision tree, and naive Bayes models exhibited greater fluctuations in AUC, reflecting lower stability. Figure 4C shows that the random forest model consistently maintained an AUC above 0.90 in the majority of samples, significantly outperforming the other models. In contrast, AUC values for naive Bayes and decision tree models mostly fell below 0.7, indicating limited generalization capability.

### 3.5 Importance of SHAP Values in Interpreting Dietary Antioxidant Properties

**FIGURE 5:**
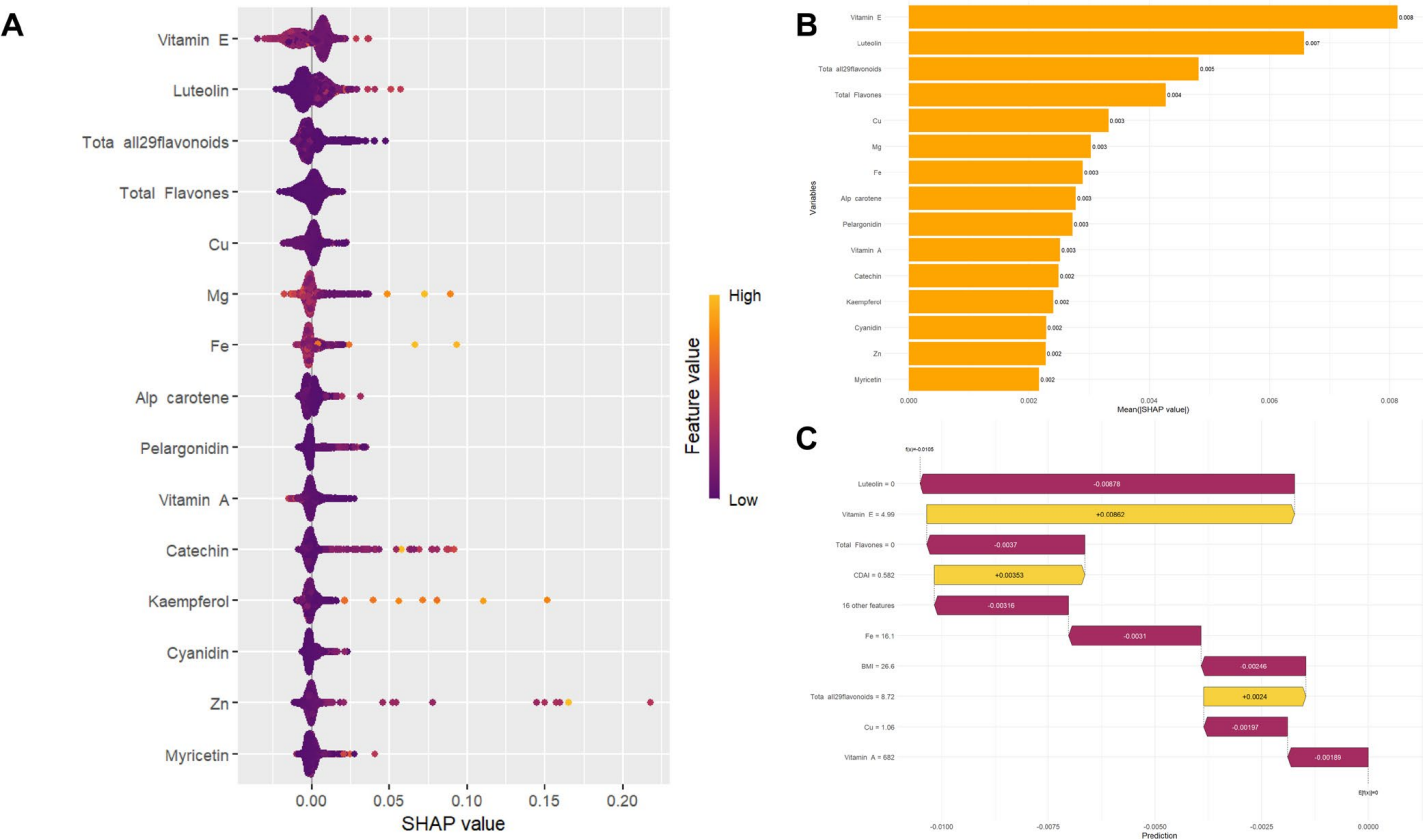
SHAP values of dietary antioxidants for random forest modeling. (A) SHAP summary plot, (B) SHAP bar graph, and (C) SHAP force diagram.

**FIGURE 6:**
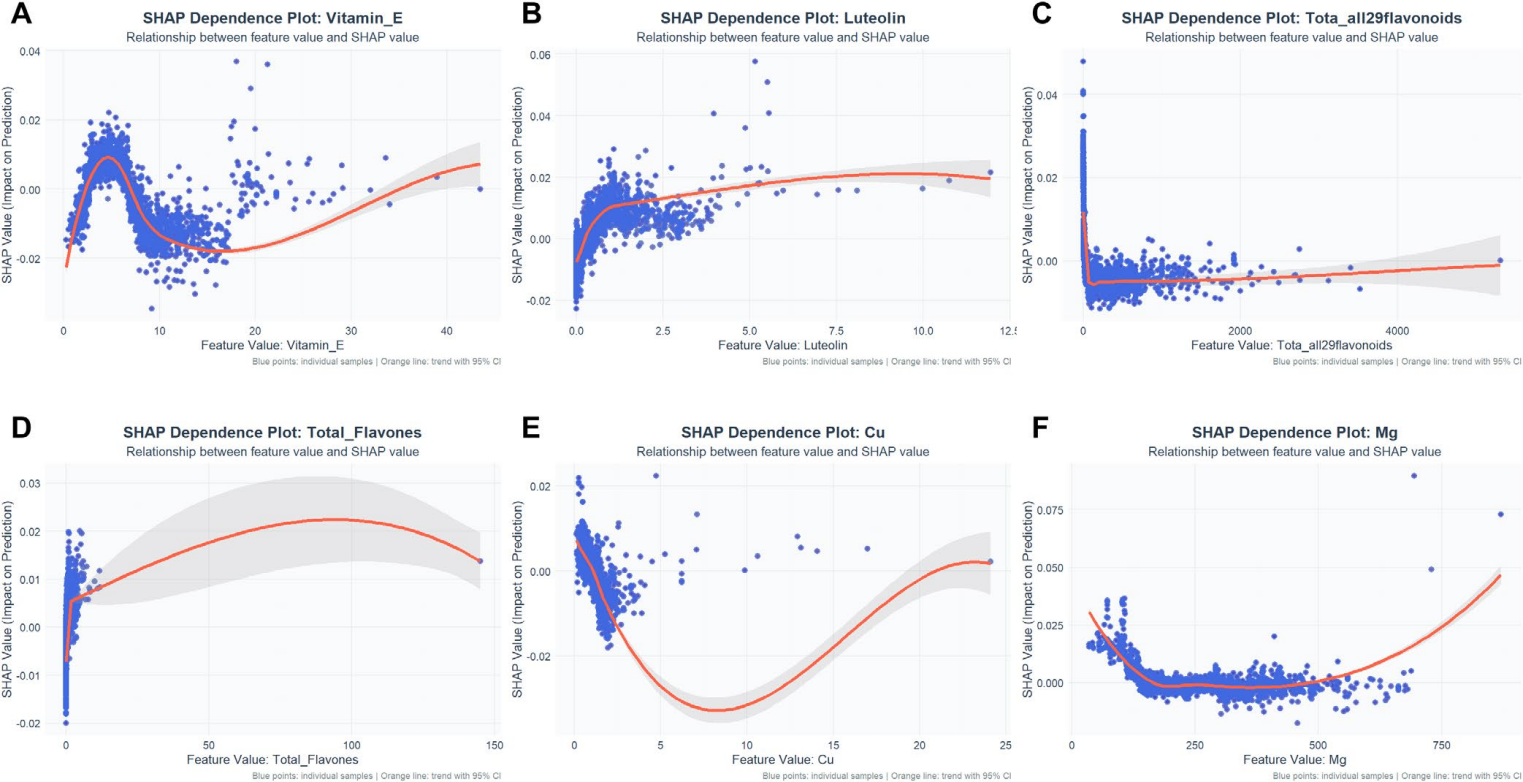
SHAP dependency plots for the top six predictor variables, sorted by SHAP importance. (A) Vitamin E, (B) Luteolin, (C) Total flavonoids, (D) Total flavones, (E) Copper (Cu), and (F) Magnesium (Mg).

The SHAP summary plot (Figure 5A) illustrates the contribution of the top 15 most important dietary antioxidant features in predicting depression risk among older adults. Based on SHAP value ranking, key predictive features included vitamin E (0.0080), luteolin (0.0070), total flavonoids (0.0066), total flavones (0.0064), copper (0.0063), magnesium (0.0061), and iron (0.0060). Most of these features had negative SHAP values, indicating an inverse association between their intake levels and depression risk. To better visualize the role of these variables in the prediction process, SHAP bar plots (Figure 5B) and force plots (Figure 5C) were generated using the shapviz package. Figure 6B presents the relative importance of dietary antioxidants in the model output, with vitamin E, luteolin, and total flavonoids showing the greatest impact. Figure 6C further demonstrates the feature effects at the individual level, where orange bars represent protective factors associated with reduced depression risk (e.g., vitamin E and luteolin), while purple bars indicate features contributing to increased risk.

Subsequently, SHAP dependence plots (Figure 6) were generated to examine the relationship between dietary antioxidant feature values and their corresponding SHAP values. The results revealed nonlinear associations for several key features, including vitamin E (Figure 6A), luteolin (Figure 6B), total flavonoids (Figure 6C), total flavones (Figure 6D), copper (Figure 6E), and magnesium (Figure 6F). In most cases, an increase in the intake of these antioxidants was associated with higher SHAP values, indicating a lower predicted risk of depression and suggesting a typical “protective effect.” Additional important features are presented in Figure S2. In addition, interaction plots between key SHAP variables and their most strongly correlated counterparts were constructed (Figure S3) to further explore feature interactions within the model. The results indicated clear synergistic effects between vitamin E and copper, luteolin and total flavonoids, and total flavones and apigenin, whereby co‐elevated levels of these nutrients were associated with a more pronounced protective effect against depression risk.

### 3.6 Online Risk Prediction Tool Development

Based on the random forest model and its top six contributing variables, an online risk prediction tool was developed (https://wdhyddx.shinyapps.io/shiny_depression_prediction_app‐1/), enhancing the model's applicability in clinical and community settings. The tool allows users to input key dietary antioxidant intake information for physically inactive adults aged 60 years and older and provides real‐time, individualized predictions of depression risk.

## 4 Discussion

Based on data from NHANES 2007–2010 and 2017–2018, this study systematically examined the relationship between dietary antioxidant intake and depression risk among physically inactive older adults, using interpretable machine learning methods. Among the six models developed, the random forest model demonstrated superior overall performance, achieving the highest accuracy (94.9%) and area under the ROC curve (AUC = 0.9425). Notably, it showed excellent sensitivity (0.9996) and good specificity (0.7181) in identifying individuals at high risk of depression. SHAP‐based interpretation further identified vitamin E, luteolin, total flavonoids, copper, magnesium, and iron as key predictors and revealed synergistic interaction effects among several feature pairs. This study not only advances the understanding of nutritional predictors for late‐life depression but also provides valuable evidence to inform early screening and dietary intervention strategies.

In the SHAP‐based interpretation, vitamin E and flavonoid‐related measures (e.g., luteolin and total flavonoids), together with redox‐related minerals (e.g., magnesium and copper), emerged among the most informative features for depression status stratification. Rather than acting in isolation [21], these nutrients likely reflect an overall antioxidant‐rich nutritional profile and may exert synergistic effects through overlapping pathways [22], including attenuation of oxidative stress and low‐grade inflammation and support of neuronal function and neuroprotection [23]. From a translational perspective, these signals are consistent with diets emphasizing minimally processed plant foods (e.g., vegetables, fruits, legumes/soy foods, nuts and seeds, and tea), which jointly contribute multiple antioxidants and cofactors involved in redox balance [24]. While our cross‐sectional design precludes causal inference, the model‐identified features provide hypothesis‐generating evidence that antioxidant‐rich dietary profiles may be informative for depression risk stratification in physically inactive older adults.

Our findings are largely consistent with those of several prospective studies. The intake of dietary antioxidants is inversely related to depression risk, which aligns with the protective effect pattern observed in our SHAP dependence plots [25, 26]. However, it is worth noting that traditionally important antioxidants such as vitamin C and β‐carotene had relatively low importance in our model, which may be attributed to the specificity of the study population. Unlike previous studies that primarily focused on single nutrients, our research revealed the synergistic effects among multiple antioxidants. SHAP interaction analysis showed significant synergistic protective effects between vitamin E and copper, and luteolin and total flavonoids. This finding supports the theory of nutrient synergy, suggesting that the combined intake of multiple antioxidants is more effective than supplementing with a single nutrient [27]. However, our results differ from some studies. Some randomized controlled trials have shown limited improvements in depressive symptoms with single antioxidant supplementation, possibly due to the neglect of nutrient interactions and individual differences in treatment response. Our machine learning approach is capable of capturing these complex nonlinear relationships, providing a more comprehensive understanding [28]. Importantly, these antioxidant signals should be interpreted within the context of whole diets, because individuals consume nutrients as part of dietary patterns rather than as isolated compounds. Several of the leading features highlighted by the model—such as vitamin E and multiple flavonoid subclasses (including flavones/flavonols and catechins)—are typically abundant in plant‐forward food groups, including leafy vegetables and herbs, citrus and other fruits, legumes/soy foods, nuts and seeds, and tea. Likewise, minerals involved in redox balance (e.g., magnesium, zinc, and copper) are often enriched in whole grains, legumes, nuts, and seafood. From a public health perspective, these findings are broadly consistent with dietary patterns characterized by higher intakes of minimally processed plant foods (e.g., Mediterranean‐style or DASH‐like patterns) and suggest that food‐based strategies—increasing fruits/vegetables, legumes/soy [29], nuts, and replacing ultra‐processed snacks with antioxidant‐rich options—may be a practical complement to depression risk stratification among physically inactive older adults. While our cross‐sectional design precludes causal inference, translating model‐identified predictors into food‐group targets may help guide hypothesis‐driven interventions and improve the interpretability and potential utility of the screening‐oriented model [13].

This study is the first to focus on physically inactive older adults aged 60 and above, a specific group with unique physiological and metabolic characteristics that highlight the protective role of dietary antioxidants. First, older adults generally experience a decline in antioxidant defense capacity. With aging, endogenous antioxidant enzyme activity decreases, leading to weakened free radical scavenging capacity and a significant increase in oxidative stress levels [30]. In this context, the supplementation of exogenous antioxidants becomes particularly important, which explains why dietary antioxidants show such high importance in our predictive model. Second, physical inactivity further amplifies the protective effects of antioxidants [31]. Regular physical activity itself has antioxidant and antidepressant effects, promoting the synthesis of endogenous antioxidant enzymes and the secretion of neurotrophic factors [32]. When this protective mechanism is lacking, dietary antioxidants serve as an important alternative for maintaining neurological health. Our SHAP analysis showed a stronger inverse relationship between antioxidant intake and depression risk in physically inactive older adults, supporting this hypothesis. Furthermore, changes in nutrient absorption and metabolism in older adults also affect the bioavailability of antioxidants. Factors such as decreased gastric acid secretion, altered gut microbiota, and drug interactions can impact the absorption efficiency of antioxidants, making it even more important to obtain various types of antioxidants from multiple food sources [33].

In recent years, machine learning methods have been widely applied to predict the risk of mental disorders, particularly depression, demonstrating superior capabilities in nonlinear modeling and variable interaction detection compared to traditional statistical models [34]. However, machine learning studies focusing on dietary antioxidant intake and depression risk remain relatively rare. Most related research has focused on behavioral and psychological factors, with limited exploration of the underlying mechanisms between nutrients and depression. While some nutritional epidemiological studies have reported associations between vitamin C, E, or flavonoids and depression levels, these studies have mostly used cross‐sectional or linear regression analyses and have yet to systematically validate their predictive performance within a machine learning framework. Furthermore, existing literature generally lacks multi‐model comparisons and performance benchmark testing, with model selection often lacking objective criteria. In contrast, this study included six mainstream algorithms, evaluated model stability using 10‐fold cross‐validation and bootstrap resampling, and incorporated SHAP analysis for interpretability of key nutrients, offering a systematic and generalizable approach for depression risk prediction.

In addition, we developed an online risk prediction tool, which can be used for self‐assessment of risk among older adults and assist primary care providers in decision‐making, thereby enhancing the translational and applied value of the model's findings. The tool is based on the random forest model and provides real‐time, individualized depression risk predictions upon input of key dietary variables, offering strong practicality and scalability.

Although the findings of this study are insightful, several limitations remain. First, the cross‐sectional nature of NHANES precludes causal inference. In particular, we cannot distinguish whether lower antioxidant intake contributes to depression risk or whether depressive symptoms lead to dietary changes that reduce antioxidant intake. Therefore, the identified predictors should be interpreted as signals useful for risk stratification/classification rather than causal determinants. Prospective cohort studies and randomized dietary interventions are needed to clarify temporality and directionality. Second, antioxidant variables were derived from NHANES 24‐h dietary recall data, which primarily reflect food and beverage intake and do not explicitly capture intake from dietary supplements. Therefore, the CDAI and OBS used here represent composite indices based on dietary intake variables rather than total (diet + supplement) antioxidant exposure. This distinction is particularly relevant in older adults, among whom supplement use is common and may substantially contribute to total micronutrient intake. In addition, antioxidants obtained from whole foods may differ from supplements in bioavailability and in their interaction with other dietary constituents (food matrix effects), and supplement use may correlate with health‐seeking behaviors and residual confounding. Moreover, although our model performed excellently in terms of predictive performance, the representativeness of the sample remains a concern. The study sample mainly comes from a specific survey database, which may introduce selection bias, and the generalizability of the results to populations from different racial, regional, and socioeconomic backgrounds requires further validation.

In summary, this study identified key nutrients, such as vitamin E, flavonoids, and magnesium, in predicting depression risk in older adults from a machine learning perspective, emphasizing the importance of antioxidant nutrition for mental health. The findings provide a precise, visual, and convenient tool for depression risk screening in older adults and offer a theoretical foundation for subsequent intervention research and the development of public health nutrition policies.

## 5 Conclusion

This study, based on machine learning and SHAP analysis, identified key dietary antioxidants, such as vitamin E, luteolin, total flavonoids, copper, magnesium, and iron, as important predictors of depression risk in physically inactive older adults, with nonlinear and synergistic interactions between variables. The developed online prediction tool offers a new approach for precision nutrition interventions. Future research should further conduct longitudinal cohort validation and mechanism exploration to strengthen the practicality and generalizability of the findings.

## Author Contributions

**Yuwen ShangGuan:** conceptualization, data curation, formal analysis, writing – original draft. **Kunpeng Wu:** investigation, software, writing – original draft. **Dong Li:** data curation, formal analysis, methodology. **Young‐Je Sim:** validation, resources. **Chuang Zhang:** methodology, writing – review and editing. **Zhenhao Lin:** data curation, formal analysis, visualization. **Litao Yan:** funding acquisition, project administration, resources, writing – review and editing.

## Funding

This work was supported by the National Natural Science Foundation of China (82202679), China Postdoctoral Science Foundation (2023M740375, 2024T170082) and Jiangsu Health International Exchange Program(2026).

## Ethics Statement

The NHANES study protocols were reviewed and approved by the National Center for Health Statistics Research Ethics Review Board. The survey cycles used in the present study were approved under NCHS ERB Protocol #2005–06 for NHANES 2007–2010 and Protocol #2018–01 for NHANES 2017–2018. Written informed consent was obtained from all participants at the time of the original NHANES data collection. More information about the NHANES database can be found at https://www.cdc.gov/nchs/nhanes/irba98.htm.

## Conflicts of Interest

The authors declare no conflicts of interest.

## Supporting information


**Figure S1:** Study subject screening flowchart.

**Figure S2:** SHAP dependency plots for the remaining 9 predictor variables not shown in the main text, among the top 15 features ranked by SHAP importance. (A) Fe, (B) Alpha‐carotene, (C) Pelargonidin, (D) Vitamin A, (E) Catechin, (F) Kaempferol, (G) Cyanidin, (H) Zn, and (I) Myricetin.

**Figure S3:** SHAP interaction plots of selected predictors in the final model.

**Table S1:** Scoring Criteria for the Composite Dietary Antioxidant Index (CDAI) and Oxidative Balance Score (OBS).

**Table S2:** Structure and scoring of the Patient Health Questionnaire‐9 (PHQ‐9).

**Table S3:** Individual dietary antioxidant intakes by depression status.

## Data Availability

The data that support the findings of this study are available from the corresponding author upon reasonable request.
