# Supplementary material for: Predicting Depression Risk in Physically Inactive Older Adults Using Dietary Antioxidants and Machine Learning: A SHAP‐Interpretable Analysis of NHANES
Source: CNS Neurosci Ther. 2026 May 30;32(6):e70961. doi: 10.1002/cns.70961 (PMC13240413; doi:10.1002/cns.70961)
Supplement: Supplementary file 1 — Figure S1: Study subject screening flowchart. [file CNS-32-e70961-s006.docx]

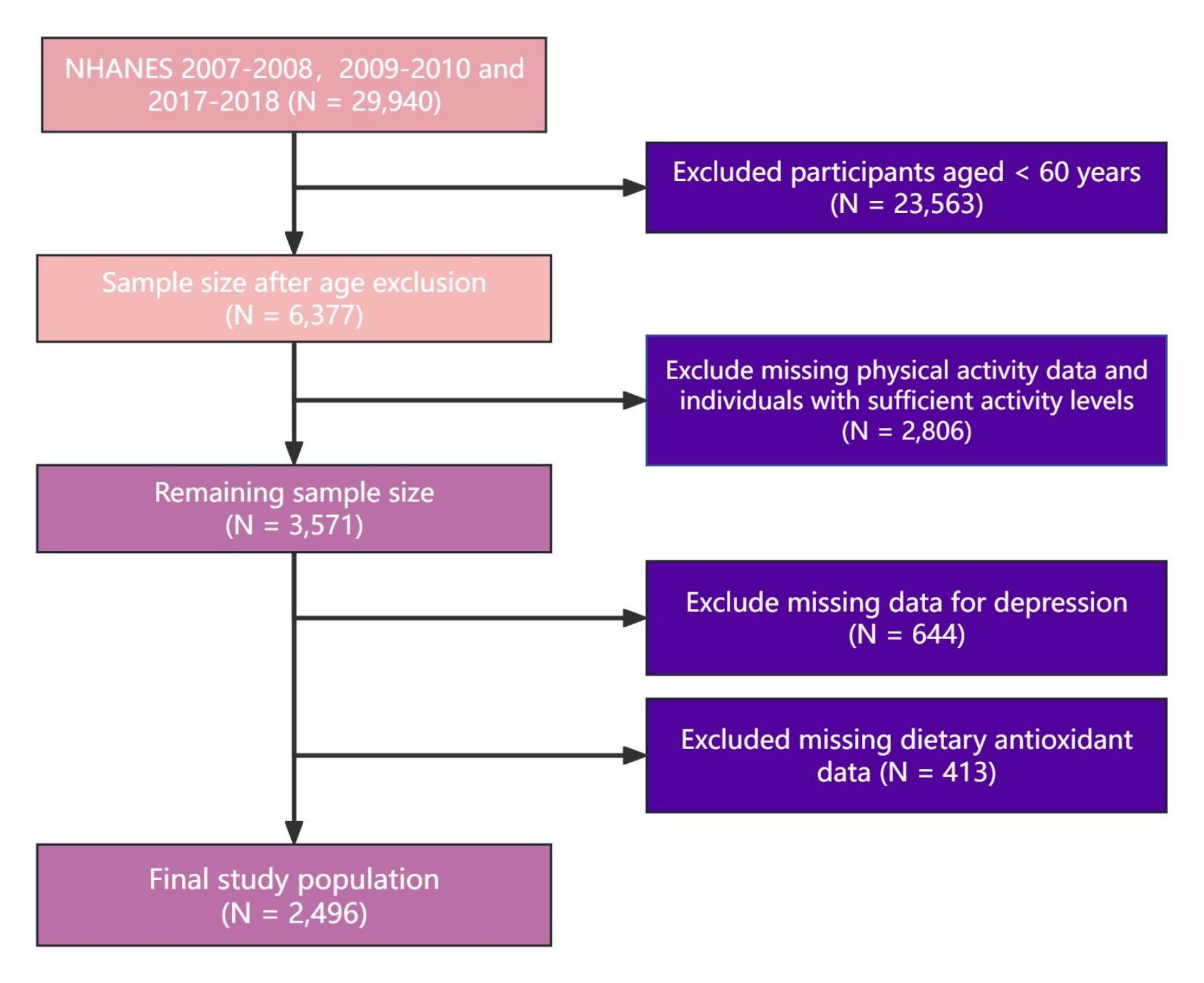


**Supplementary Figure 1.** Study subject screening flowchart

Flowchart showing sample selection from NHANES 2007–2010 and 2017–2018. The initial sample included 29,940 participants. Exclusions were: age <60 years (n=23,563), adequate physical activity (n=2,806; defined as ≥600 MET-min/week), missing depression data (n=644), and missing dietary antioxidant data (n=431). The final analytical sample included 2,496 physically inactive adults aged ≥60 years.
