# Supplementary material for: Predicting Depression Risk in Physically Inactive Older Adults Using Dietary Antioxidants and Machine Learning: A SHAP‐Interpretable Analysis of NHANES
Source: CNS Neurosci Ther. 2026 May 30;32(6):e70961. doi: 10.1002/cns.70961 (PMC13240413; doi:10.1002/cns.70961)
Supplement: Supplementary file 2 — Figure S2: SHAP dependency plots for the remaining 9 predictor variables not shown in the main text, among the top 15 features ranked by SHAP importance. (A) Fe, (B) Alpha‐carotene, (C) Pelargonidin, (D) Vitamin A, (E) Catechin, (F) Kaempferol, (G) Cyanidin, (H) Zn, and (I) Myricetin. [file CNS-32-e70961-s002.docx]

**
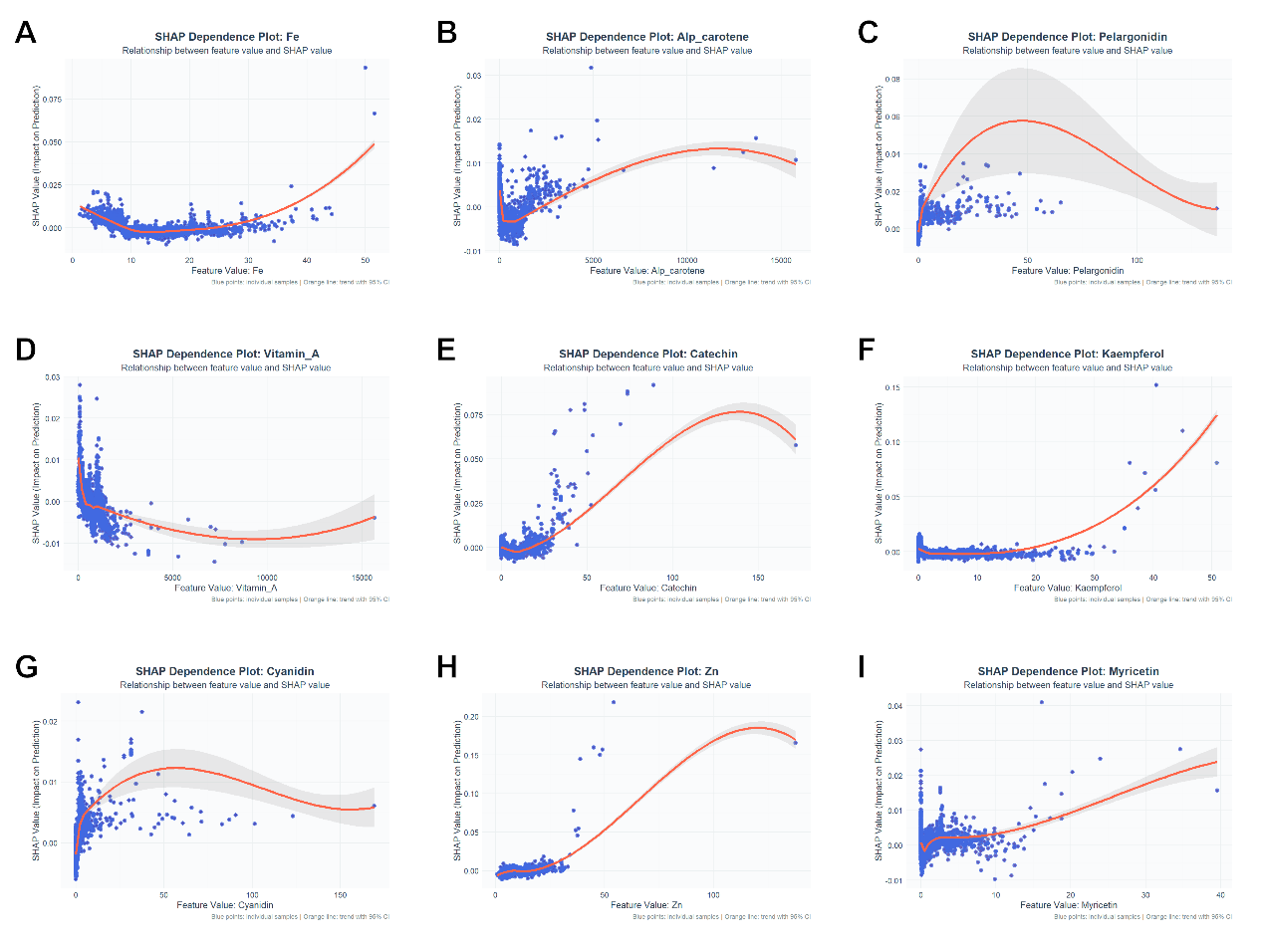
**

**Supplementary Figure 2.** SHAP dependency plots for the remaining 9 predictor variables not shown in the main text, among the top 15 features ranked by SHAP importance. (A) Fe, (B) Alpha-carotene, (C) Pelargonidin, (D) Vitamin A, (E) Catechin, (F) Kaempferol, (G) Cyanidin, (H) Zn, and (I) Myricetin.

Each plot shows the association between feature values and SHAP values, reflecting the contribution of each predictor to depression risk prediction among physically inactive older adults.
