# Supplementary material for: Predicting Depression Risk in Physically Inactive Older Adults Using Dietary Antioxidants and Machine Learning: A SHAP‐Interpretable Analysis of NHANES
Source: CNS Neurosci Ther. 2026 May 30;32(6):e70961. doi: 10.1002/cns.70961 (PMC13240413; doi:10.1002/cns.70961)
Supplement: Supplementary file 3 — Figure S3: SHAP interaction plots of selected predictors in the final model. [file CNS-32-e70961-s003.docx]

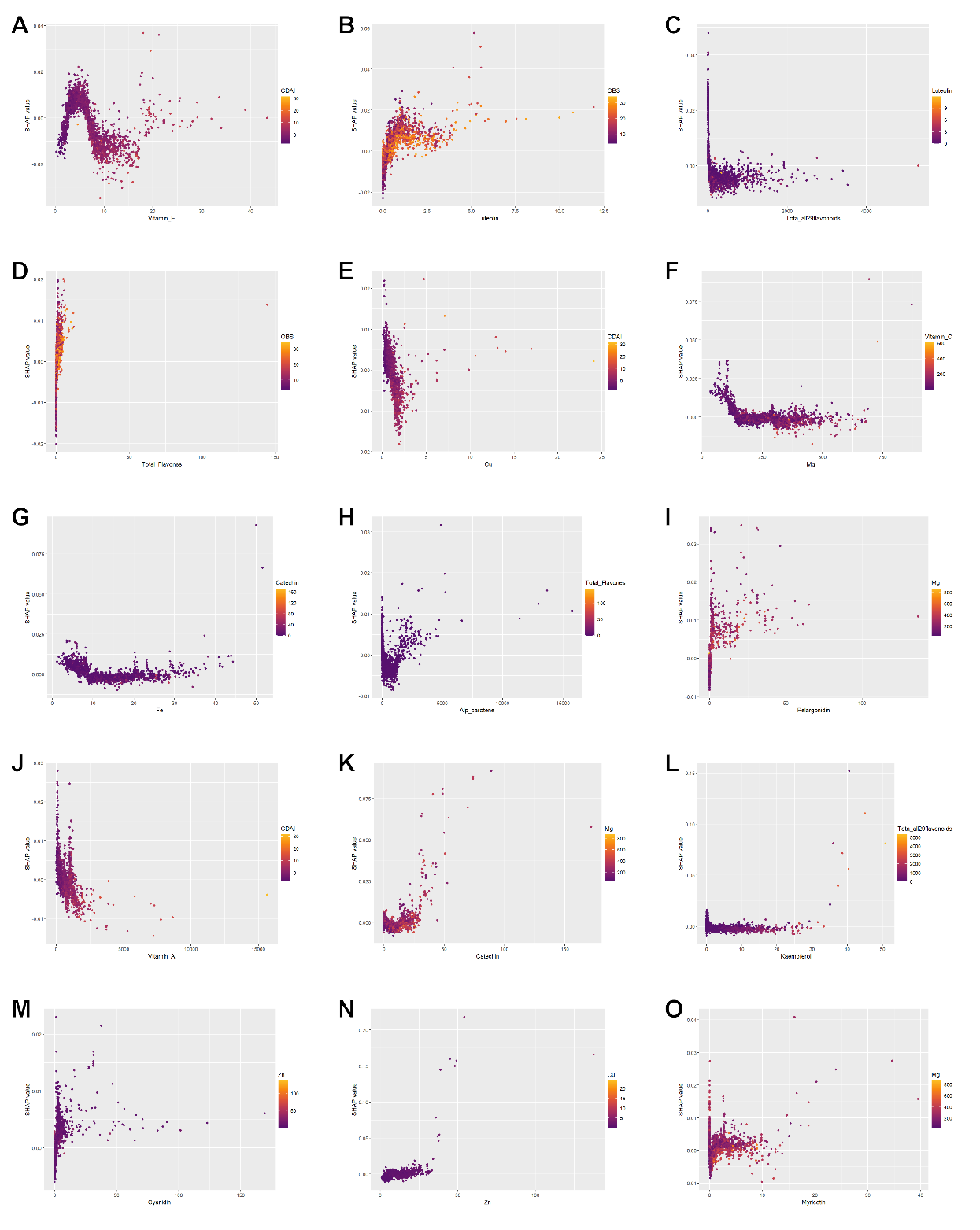


**Supplementary Figure 3.** SHAP interaction plots of selected predictors in the final model.

A-O Each plot shows the interaction effect between two selected dietary antioxidant-related predictors and their contribution to depression risk prediction. The x-axis represents the feature value, the y-axis represents the SHAP interaction value, and the color gradient indicates the value of the interacting feature. Positive values indicate increased contribution to the predicted risk, whereas negative values indicate decreased contribution.
