# Supplementary material for: Predicting Depression Risk in Physically Inactive Older Adults Using Dietary Antioxidants and Machine Learning: A SHAP‐Interpretable Analysis of NHANES
Source: CNS Neurosci Ther. 2026 May 30;32(6):e70961. doi: 10.1002/cns.70961 (PMC13240413; doi:10.1002/cns.70961)
Supplement: Supplementary file 4 — Table S1: Scoring Criteria for the Composite Dietary Antioxidant Index (CDAI) and Oxidative Balance Score (OBS). [file CNS-32-e70961-s005.docx]

**Supplementary Table 1**

1. Composite Dietary Antioxidant Index (CDAI) [1]

| **Component** | **Details** |
| --- | --- |
| Formula | CDAI = Σ(xi - μi)/Si |
| Variables | xi = daily intake of each antioxidant for individual i<br>μi = sex-specific mean intake for each antioxidant<br>Si = sex-specific standard deviation for each antioxidant |
| Antioxidant Components | Vitamin A (μg RE/day), Vitamin C (mg/day), Vitamin E (mg/day), Zinc (mg/day), Selenium (μg/day), Carotenoids (μg/day) - sum of α-carotene, β-carotene, and carotene RE |
| Interpretation | Higher scores = greater dietary antioxidant capacity<br>Standardized: mean = 0, SD = 1 <br>Positive scores = above-average antioxidant intake |

2.Oxidative balance score assignment scheme[2]

| OBS components | Property | Male | | | Female | | |
| --- | --- | --- | --- | --- | --- | --- | --- |
|  |  | 0 | 1 | 2 | 0 | 1 | 2 |
| **Dietary OBS components** | | | | | | | |
| Dietary fiber (g/d) | antioxidant | <12.56 | 12.56-19.70 | ≥19.70 | <10.10 | 10.10-16.31 | ≥16.31 |
| Carotene (RE/d) | antioxidant | <98.83 | 98.83-306.25 | ≥306.25 | <98.08 | 98.08-383.50 | ≥383.50 |
| Riboflavin (mg/d) | antioxidant | <1.79 | 1.79-2.69 | ≥2.69 | <1.34 | 1.34-2.02 | ≥2.02 |
| Niacin (mg/d) | antioxidant | <20.65 | 20.65-29.75 | ≥29.75 | <14.52 | 14.52-21.86 | ≥21.86 |
| Vitamin B₆ (mg/d) | antioxidant | <1.59 | 1.59-2.40 | ≥2.40 | <1.13 | 1.13-1.77 | ≥1.77 |
| Total folate (mcg/d) | antioxidant | <316.00 | 316.00-492.00 | ≥492.00 | <251.00 | 251.00-388.96 | ≥388.96 |
| Vitamin B₁₂ (mcg/d) | antioxidant | <3.36 | 3.36-6.20 | ≥6.20 | <2.22 | 2.22-4.22 | ≥4.22 |
| Vitamin C (mg/d) | antioxidant | <42.44 | 42.44-113.21 | ≥113.21 | <38.01 | 38.01-98.49 | ≥98.49 |
| Vitamin E (ATE) (mg/d) | antioxidant | <5.82 | 5.82-9.42 | ≥9.42 | <4.53 | 4.53-7.52 | ≥7.52 |
| Calcium (mg/d) | antioxidant | <646.00 | 646.00-1072.00 | ≥1072.00 | <499.24 | 499.24-849.00 | ≥849.00 |
| Magnesium (mg/d) | antioxidant | <257.00 | 257.00-361.28 | ≥361.28 | <187.00 | 187.00-283.43 | ≥283.43 |
| Zinc (mg/d) | antioxidant | <9.75 | 9.75-15.10 | ≥15.10 | <6.73 | 6.73-10.75 | ≥10.75 |
| Copper (mg/d) | antioxidant | <1.12 | 1.12-1.57 | ≥1.57 | <0.85 | 0.85-1.28 | ≥1.28 |
| Selenium (mcg/d) | antioxidant | <94.94 | 94.94-141.80 | ≥141.80 | <67.79 | 67.79-99.50 | ≥99.50 |
| Total fat (g/d) | prooxidant | ≥69.83 | 69.83-107.43 | <107.43 | ≥75.79 | 50.98-75.79 | <50.98 |
| Iron (mg/d) | prooxidant | ≥12.88 | 12.88-19.17 | <19.17 | ≥9.65 | 9.65-14.32 | <14.32 |
| **Lifestyle OBS components** | | | | | | | |
| Physical activity (MET-minute/week) | antioxidant | <417.86 | 417.86-1135.71 | ≥1135.71 | <270.00 | 270.00-845.71 | ≥845.71 |
| Alcohol (g/d) | prooxidant | ≥30 | 0-30 | None | ≥15 | 0-15 | None |
| Body mass index (kg/m²) | prooxidant | ≥25.54 | 25.54-29.17 | <29.17 | ≥23.74 | 23.74-28.64 | <28.64 |
| Cotinine (ng/mL) | prooxidant | ≥0.038 | 0.038-1.13 | <1.13 | ≥0.035 | 0.035-0.172 | <0.172 |

OBS: oxidative balance score; RE: retinol equivalent; ATE: alpha-tocopherol equivalent; MET: metabolic equivalent.

References

1. Wright ME, Mayne ST, Stolzenberg-Solomon RZ, Li Z, Pietinen P, Taylor PR, et al. Development of a comprehensive dietary antioxidant index and application to lung cancer risk in a cohort of male smokers. Am J Epidemiol [Internet]. 2004 [cited 2025 Dec 31];160:68–76. https://doi.org/10.1093/aje/kwh173

2. Qu H. The association between oxidative balance score and periodontitis in adults: A population-based study. Front Nutr. 2023;10:1138488. https://doi.org/10.3389/fnut.2023.1138488
