# Supplementary material for: Predicting Depression Risk in Physically Inactive Older Adults Using Dietary Antioxidants and Machine Learning: A SHAP‐Interpretable Analysis of NHANES
Source: CNS Neurosci Ther. 2026 May 30;32(6):e70961. doi: 10.1002/cns.70961 (PMC13240413; doi:10.1002/cns.70961)
Supplement: Supplementary file 5 — Table S2: Structure and scoring of the Patient Health Questionnaire‐9 (PHQ‐9). [file CNS-32-e70961-s004.docx]

**Supplementary Table 2.** Structure and scoring of the Patient Health Questionnaire-9 (PHQ-9)

| Item | Question content (abbreviated) | Response options | Score |
| --- | --- | --- | --- |
| PHQ-1 | Little interest or pleasure in doing things | Not at all / Several days / More than half the days / Nearly every day | 0–3 |
| PHQ-2 | Feeling down, depressed, or hopeless | 0–3 |  |
| PHQ-3 | Trouble falling or staying asleep, or sleeping too much | 0–3 |  |
| PHQ-4 | Feeling tired or having little energy | 0–3 |  |
| PHQ-5 | Poor appetite or overeating | 0–3 |  |
| PHQ-6 | Feeling bad about yourself | 0–3 |  |
| PHQ-7 | Trouble concentrating | 0–3 |  |
| PHQ-8 | Moving or speaking slowly / being fidgety | 0–3 |  |
| PHQ-9 | Thoughts of self-harm | 0–3 |  |

Total PHQ-9 scores range from 0 to 27, with higher scores indicating more severe depressive symptoms. A cutoff of ≥10 was used to define depression in this study.
