# Supplementary material for: Predicting Depression Risk in Physically Inactive Older Adults Using Dietary Antioxidants and Machine Learning: A SHAP‐Interpretable Analysis of NHANES
Source: CNS Neurosci Ther. 2026 May 30;32(6):e70961. doi: 10.1002/cns.70961 (PMC13240413; doi:10.1002/cns.70961)
Supplement: Supplementary file 6 — Table S3: Individual dietary antioxidant intakes by depression status. [file CNS-32-e70961-s001.docx]

**Supplementary Table 3.** Individual dietary antioxidant intakes by depression status.

| Variable | Non- Depression  (N=2275) | Depression  (N=221) | p-value |
| --- | --- | --- | --- |
| Alp carotene, µg/day | 403.21 (831.20) | 323.16 (608.93) | 0.163 |
| Beta carotene, µg/day | 2216.83 (2908.82) | 1775.87 (2198.75) | 0.028 |
| Carotene Retinol Equivalent, µg/day | 201.54 (265.13) | 161.45 (201.33) | 0.029 |
| Vitamin A, µg/day | 623.16 (628.26) | 572.75 (419.93) | 0.243 |
| Vitamin C, mg/day | 77.29 (64.76) | 64.35 (59.41) | 0.004 |
| Vitamin E, mg/day | 6.75 (4.09) | 6.06 (3.69) | 0.016 |
| Vitamin D, µg/day | 4.45 (6.29) | 4.88 (5.21) | 0.121 |
| Mg, mg/day | 249.73 (100.30) | 240.12 (98.14) | 0.173 |
| Fe, mg/day | 13.07 (6.28) | 11.97 (5.34) | 0.013 |
| Zn, mg/day | 9.62 (5.64) | 9.05 (4.55) | 0.140 |
| Cu, mg/day | 1.12 (0.94) | 1.03 (0.60) | 0.180 |
| Se, µg/day | 92.81 (41.79) | 87.96 (39.20) | 0.098 |
| Daidzein, mg/day | 0.52 (5.35) | 0.43 (2.11) | 0.794 |
| Genistein, mg/day | 0.70 (7.19) | 0.66 (3.48) | 0.936 |
| Glycitein, mg/day | 0.10 (1.19) | 0.10 (0.62) | 0.985 |
| Cyanidin, mg/day | 2.16 (7.95) | 1.36 (3.76) | 0.137 |
| Petunidin, mg/day | 0.93 (5.38) | 0.49 (2.03) | 0.219 |
| Delphinidin, mg/day | 1.22 (6.85) | 0.69 (2.56) | 0.253 |
| Malvidin, mg/day | 3.87 (15.07) | 2.85 (9.29) | 0.324 |
| Pelargonidin, mg/day | 1.48 (6.42) | 0.84 (3.73) | 0.145 |
| Peonidin, mg/day | 1.46 (7.95) | 0.48 (1.47) | 0.067 |
| Catechin, mg/day | 6.72 (8.57) | 6.22 (8.60) | 0.410 |
| Epigallocatechin, mg/day | 13.83 (41.23) | 13.38 (36.47) | 0.876 |
| Epicatechin, mg/day | 8.46 (13.47) | 7.87 (12.43) | 0.531 |
| Epicatechin 3 gallate, mg/day | 8.95 (26.68) | 8.46 (23.36) | 0.792 |
| Epigallocatechin 3 gallate, mg/day | 24.05 (86.07) | 23.75 (73.72) | 0.960 |
| Theaflavin, mg/day | 1.32 (3.64) | 1.13 (3.28) | 0.468 |
| Thearubigins, mg/day | 78.53 (198.83) | 75.10 (192.02) | 0.806 |
| Eriodictyol, mg/day | 0.14 (0.83) | 0.14 (0.89) | 0.983 |
| Hesperetin, mg/day | 9.48 (22.25) | 9.31 (25.10) | 0.916 |
| Naringenin, mg/day | 3.51 (10.88) | 4.01 (13.25) | 0.529 |
| Apigenin, mg/day | 0.23 (2.95) | 0.12 (0.26) | 0.611 |
| Luteolin, mg/day | 0.54 (0.92) | 0.46 (0.69) | 0.169 |
| Isorhamnetin, mg/day | 0.69 (1.13) | 0.67 (1.25) | 0.806 |
| Kaempferol, mg/day | 3.51 (5.11) | 3.25 (5.57) | 0.478 |
| Myricetin, mg/day | 1.37 (2.45) | 1.44 (2.98) | 0.701 |
| Quercetin, mg/day | 9.81 (10.19) | 9.19 (11.16) | 0.395 |
| Theaflavin 3 digallate, mg/day | 1.46 (4.02) | 1.25 (3.62) | 0.467 |
| Theaflavin 3q gallate, mg/day | 1.22 (3.45) | 1.04 (3.11) | 0.466 |
| Theaflavin 3 gallate, mg/day | 1.06 (2.89) | 0.91 (2.60) | 0.475 |
| Gallocatechin, mg/day | 1.37 (3.65) | 1.23 (3.27) | 0.582 |
| Subtotal Catechins, mg/day | 63.38 (174.37) | 60.92 (152.74) | 0.839 |
| Total Isoflavones, mg/day | 1.32 (13.71) | 1.18 (6.15) | 0.884 |
| Total Anthocyanidins, mg/day | 11.13 (33.89) | 6.71 (15.77) | 0.055 |
| Total Flavan 3 ols, mg/day | 146.96 (347.39) | 140.36 (331.87) | 0.787 |
| Total Flavanones, mg/day | 13.13 (30.56) | 13.46 (33.83) | 0.881 |
| Total Flavones, mg/day | 0.77 (3.21) | 0.58 (0.80) | 0.383 |
| Total Flavonols, mg/day | 15.37 (16.78) | 14.55 (18.57) | 0.489 |
| Total Sum 29 flavonoids, mg/day | 188.69 (365.04) | 176.84 (351.20) | 0.644 |

Continuous variables were presented as medians and standard deviations (SD)
